# A description of novel variants and review of phenotypic spectrum in *UBA5*-related early epileptic encephalopathy

**DOI:** 10.1101/mcs.a005827

**Published:** 2021-06

**Authors:** Lauren C. Briere, Melissa A. Walker, Frances A. High, Cynthia Cooper, Cassandra A. Rogers, Christine J. Callahan, Ryosuke Ishimura, Yoshinobu Ichimura, Paul A. Caruso, Nutan Sharma, Elly Brokamp, Mary E. Koziura, Shekeeb S. Mohammad, Russell C. Dale, Lisa G. Riley, Undiagnosed Diseases Network, John A. Phillips, Masaaki Komatsu, David A. Sweetser

**Affiliations:** 1 Department of Pediatrics, Division of Medical Genetics and Metabolism, and Center for Genomic Medicine, Massachusetts 02114, USA;; 2 Department of Neurology, Division of Neurogenetics, Child Neurology, Massachusetts 02114, USA;; 3 Department of Pediatrics, Division of Medical Genetics and Metabolism, Massachusetts 02114, USA;; 4 Department of Internal Medicine, Massachusetts General Hospital, Boston, Massachusetts 02114, USA;; 5 Department of Medicine, Brigham and Women's Hospital, Boston, Massachusetts 02115, USA;; 6 Department of Biochemistry, Niigata University School of Medical and Dental Sciences, Chuo-ku, Niigata 951-8510, Japan;; 7 Department of Radiology, Massachusetts General Hospital, Boston, Massachusetts 02114, USA;; 8 Department of Neurology, Massachusetts General Hospital, Boston, Massachusetts 02114, USA;; 9 Division of Medical Genetics and Genomic Medicine, Vanderbilt University School of Medicine, Nashville, Tennessee 37232, USA;; 10 Kids Neuroscience Center & Children's Hospital at Westmead Clinical School, The University of Sydney, Sydney, New South Wales 2006, Australia;; 11 Rare Diseases Functional Genomics, Kids Research, The Children's Hospital at Westmead and Children's Medical Research Institute, Westmead, New South Wales 2145, Australia;; 12 Discipline of Child & Adolescent Health, University of Sydney, Sydney, New South Wales 2006, Australia;; 13 Department of Physiology, Juntendo University School of Medicine, Tokyo 113-8421, Japan;; 15 Undiagnosed Disease Program, NIH Bethesda, Maryland 20892, USA;; 16 National Human Genome Research Institute, Bethesda, Maryland 20892, USA;; 17 Boston Children's Hospital, Boston, Massachusetts 02115, USA;; 18 Baylor College of Medicine, Houston, Texas 77030, USA;; 19 University of Utah, Salt Lake City, Utah 84112, USA;; 20 University of Washington School of Medicine, Seattle, Washington 98195, USA;; 21 Stanford Medicine, Stanford, California 94305, USA;; 22 University of Miami School of Medicine, Miami, Florida 33136, USA;; 23 Washington University, Saint Louis, Missouri 63130, USA;; 24 Washington University Model Organisms Screening Center, Saint Louis, Missouri 63110, USA;; 25 University of Utah/ARUP, Salt Lake City, Utah 84108, USA;; 26 Children's Hospital of Philadelphia, Philadelphia, Pennsylvania 19104, USA;; 27 Vanderbilt University, Nashville, Tennessee 37235, USA;; 28 Massachusetts General Hospital, Boston, Massachusetts 02114, USA;; 29 University of California at Los Angeles, Los Angeles, California 90095, USA;; 30 University of Alabama at Birmingham, Birmingham, Alabama 35294, USA;; 31 Brigham and Women's Hospital, Boston, Massachusetts 02115, USA;; 32 National Institutes of Health, Bethesda, Maryland 20892, USA;; 33 Duke University, Durham, North Carolina 27708, USA;; 34 Mayo Clinic, Rochester, Minnesota 55902, USA;; 35 Baylor Medical Genetics Laboratories, Houston, Texas 77021, USA;; 36 Harvard Medical School, Boston, Massachusetts 02115, USA;; 37 Columbia University, New York, New York 10027, USA;; 38 Baylor College of Medicine, Model Organisms Screening Center, Houston, Texas 77030, USA;; 39 Institute of Neuroscience, University of Oregon, Eugene, Oregon 97403, USA;

**Keywords:** central hypotonia, epileptic encephalopathy, generalized dystonia, limb hypertonia, profound global developmental delay, severe global developmental delay

## Abstract

Early infantile epileptic encephalopathy-44 (EIEE44, MIM: 617132) is a previously described condition resulting from biallelic variants in *UBA5*, a gene involved in a ubiquitin-like post-translational modification system called UFMylation. Here we report five children from four families with biallelic pathogenic variants in *UBA5*. All five children presented with global developmental delay, epilepsy, axial hypotonia, appendicular hypertonia, and a movement disorder, including dystonia in four. Affected individuals in all four families have compound heterozygous pathogenic variants in *UBA5*. All have the recurrent mild c.1111G > A (p.Ala371Thr) variant in *trans* with a second *UBA5* variant. One patient has the previously described c.562C > T (p. Arg188*) variant, two other unrelated patients have a novel missense variant, c.907T > C (p.Cys303Arg), and the two siblings have a novel missense variant, c.761T > C (p.Leu254Pro). Functional analyses demonstrate that both the p.Cys303Arg variant and the p.Leu254Pro variants result in a significant decrease in protein function. We also review the phenotypes and genotypes of all 15 previously reported families with biallelic *UBA5* variants, of which two families have presented with distinct phenotypes, and we describe evidence for some limited genotype–phenotype correlation. The overlap of motor and developmental phenotypes noted in our cohort and literature review adds to the increasing understanding of genetic syndromes with movement disorders-epilepsy.

## INTRODUCTION

Post-translational protein modification, including ubiquitination and ubiquitin-like modification, plays crucial roles in many cellular processes such as control of cell cycle, protein–protein interactions, signal transduction pathways, cellular differentiation, and apoptosis (Wei and Xu 2016). Together, *UBA5* [MIM: 610552], *UFM1* [MIM: 610553], *UFC1* [MIM: 610554], *UFL1* [MIM: 613372], and *DDRGK1* [MIM:616177] encode proteins that comprise a ubiquitin-like post-translational modification system termed UFMylation. All five genes are ubiquitously expressed and conserved in metazoa and plants, implying an important role in regulating cellular function (Komatsu et al. 2004; Sasakawa et al. 2006; Tatsumi et al. 2010; Cai et al. 2015; Muona et al. 2016).

**Figure 1.**
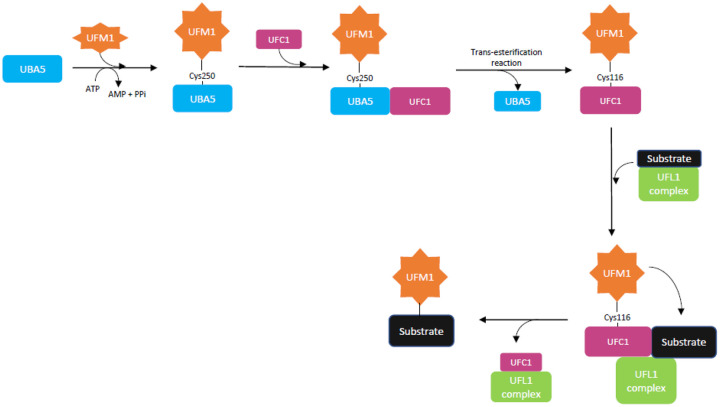
In the first step of UFMylation, UFM1 is activated by UBA5 (E1-like enzyme) and forms a thioester bond with the Cys250 residue of UBA5. In the second step, UFC1 (E2-like enzyme) binds to UBA5, and UFM1 is transferred to the Cys116 residue of UFC1 by a *trans*-esterification reaction. In the third step, the UFL1 complex (E3-like enzyme) brings a substrate to the UFC1–UFM1 complex, and UFM1 is covalently conjugated to the substrate. The functional studies performed in this study measure the presence of the UFM1–UBA5 and UFM1–UFC1 conjugates.

UFMylation is a three-step process by which UFM1 is conjugated to target proteins (Fig. 1). UBA5, an E1 (activating) enzyme that forms a homodimer, is involved in the first two steps (Komatsu et al. 2004; Tatsumi et al. 2010; Oweis et al. 2016; Wei and Xu 2016). In the first step, UFM1 is activated by UBA5 and forms a high-energy thioester bond with the cysteine (Cys) 250 reside within the catalytic domain of UBA5. In the second step the E2 (conjugating) enzyme, UFC1, binds to the carboxy-terminal domain of UBA5, and the activated UFM1 is transferred to the Cys116 residue of UFC1 by a *trans*-esterification reaction. Finally, the UFM1 specific E3 (ligating) enzyme complex, consisting of UFL1 and DDRGK1, brings a substrate to the UFC1–UFM1 complex, and UFM1 is conjugated to the substrate (Oweis et al. 2016).

The functions of UFMylation have not yet been fully elucidated and relatively few targets have been identified, but recent studies have shown that UFMylation has important roles in proteostasis control and lipid metabolism (Gannavaram et al. 2012; Gerakis et al. 2019; Yang et al. 2019). Multiple studies indicate UFMylation is involved in maintaining endoplasmic reticulum homeostasis, and recent studies have specified the role of UFMylation in endoplasmic reticulum (ER)-phagy and in maintaining ER translocation function under high secretory conditions via a noncanonical mechanism (Azfer et al. 2006; Lemaire et al. 2011; Tatsumi et al. 2011; Zhang et al. 2012, 2015; Gerakis et al. 2019; Walczak et al. 2019; Liang et al. 2020; Stephani et al. 2020; Wang et al. 2020). Dysfunction of UFMylation and its targets has been reported in association with hematologic disease, breast cancer, schizophrenia, ischemic heart disease, and diabetes (Azfer et al. 2006; Lemaire et al. 2011; Yoo et al. 2014; Cai et al. 2015; Wei and Xu 2016).

Cases of human germline variation in *UBA5* were first described in 2016 (Colin et al. 2016; Duan et al. 2016; Muona et al. 2016). To date, what appear to be three different *UBA5*-associated autosomal recessive disorders have been described.

Early infantile epileptic encephalopathy-44 (EEIE44) (MIM: 617132) is an autosomal recessive condition resulting from biallelic *UBA5* variants that is characterized by early-onset encephalopathy with or without seizures, as well as abnormal tone, movement abnormalities, severe global developmental delay, and intellectual disability. Many but not all previously reported cases have visual impairment, and many have significant failure to thrive and acquired microcephaly, often despite adequate caloric intake. Variable nonspecific brain magnetic resonance imaging (MRI) findings, such as delayed myelination, thinning of the corpus callosum, cerebral and cerebellar atrophy, and white matter hyperintensities, were reported in most patients studied. In many patients, imaging was normal in the first months of life but later showed abnormalities. A total of 19 children from 13 families have been previously reported in the literature beginning in 2016 (Colin et al. 2016; Muona et al. 2016; Arnadottir et al. 2017; Daida et al. 2018; Mignon-Ravix et al. 2018; Low et al. 2019).

Duan et al. and Cabrera-Serrano et al. have described *UBA5*-associated phenotypes that are distinct from early infantile epileptic encephalopathy-44 (EIEE44). Biallelic *UBA5* mutations were first reported by Duan et al. (2016), who reported two siblings with a progressive childhood-onset cerebellar ataxia with normal intellect, listed in OMIM as spinocerebellar ataxia, autosomal recessive 24 (MIM: 617133). Most recently, Cabrera-Serrano et al. (2020) described a large consanguineous family in which five affected individuals had a severe congenital neuropathy that resulted in death in early infancy due to respiratory failure.

Here we describe an additional five patients from four families with phenotypes consistent with EIEE44. All five patients carry the previously described hypomorphic p.Ala371Thr variant in *trans* with a second *UBA5* variant. We describe two novel *UBA5* variants and present functional data supporting their pathogenicity. Last, we review the phenotypic and genotypic information of all previously described patients with biallelic *UBA5* variants and the available functional data to date.

## RESULTS

### Clinical Presentations

Patient 1 is an 8-yr-old female with profound global developmental delay, cortical visual impairment, seizures, axial hypotonia, appendicular hypertonia and dystonia, and failure to thrive. Although she initially had no history of seizures, electroencephalogram (EEG) showed spikes and polyspikes occurring virtually every 10 sec with background notable for slowing and disorganization. Because of this finding, the patient had been on prophylactic antiepileptic medications since infancy. EEGs at 3 and 4.5 yr showed multifocal epileptiform discharges without clear clinical correlate. The patient began having clinical seizures at 5.75 yr of age, and an EEG at 6 yr of age captured generalized tonic-clonic seizures. Her seizures are drug-resistant on a combination of clonazepam, zonisamide, clobazam, and cannabidiol oil. Brain imaging was normal at 5 mo but showed mild, nonspecific abnormalities by 22 mo: posterior thinning of the corpus callosum, patchy T2 signal prolongation in the white matter, and incompletely rotated hippocampi. The patient does not have microcephaly, but her head circumference is less than expected for her family (patient 48th percentile, parents 98th and 99th percentiles).

Patient 2 is a 7-yr-old female with severe global developmental delay, failure to thrive, mild microcephaly (−2.0 SD), axial hypotonia, appendicular hypertonia and spasticity, and a movement disorder consisting of dystonia and chorea. She has mild esotropia and right amblyopia. The patient began having episodes concerning for seizures at 5 yr of age. An EEG did not capture any clinical events but showed a photoparoxysmal response in the form of bilateral occipital sharp waves with bilateral synchrony. The patient was started on levetiracetam and has had no further episodes. The patient's chorea was described as mild and resolved with clonazepam. The patient's dystonia was severe despite multiple medications. A deep brain stimulator was placed in the bilateral globus pallidus at 7 yr of age, resulting in dramatic improvement in her dystonia and subsequent developmental gains in all domains. Brain MRI at 10 mo showed that the left hippocampal head was mildly smaller than the right, without signal asymmetry. This was thought to represent normal variation versus possible mesial temporal sclerosis. Subsequent brain MRIs at 30 mo and 5 yr were essentially unchanged.

Patient 3 is 19-yr-old male with global developmental delay, intellectual disability, epilepsy, axial hypotonia, appendicular hypertonia, ataxia, strabismus, failure to thrive, and microcephaly. He was diagnosed with partial complex and generalized tonic-clonic seizures at 4 yr of age. An EEG at 7 yr of age showed diffuse delta slowing intermixed with α background; consistent with mild, diffuse cerebral dysfunction. The frequency of his seizures increased at 16 yr of age, and at that time he experienced drug-resistant generalized atonic, tonic-clonic, and absence seizures. His seizures are currently well-controlled on lamotrigine and brivaracetam, with no seizures within the past 2 years. Patient 3 had brain MRIs at 3 yr and at 8 yr, which were reported as normal, whereas a brain MRI at 15 yr noted microcephaly and decreased frontal white matter parenchymal volume.

Patient 4, the younger sister of Patient 3, is a 16-yr-old female with global developmental delay, intellectual disability, drug-resistant multifocal and generalized epilepsy, hypotonia, mild spasticity, dystonia, failure to thrive, microcephaly, astigmatism, and myopia. Infantile spasms were first noted at 7 mo. An EEG at 9 mo of age showed extremely frequent bifrontal predominant high-voltage 2- to 3-Hz epileptiform discharges. An EEG at 2 yr of age was consistent with mild to moderate generalized encephalopathy. Despite a vagus nerve stimulator, the patient continues to have multiple seizures per day, which include generalized myoclonic, myoclonic-atonic, myoclonic-tonic-clonic, and focal to bilateral tonic-clonic seizures. She is nonambulatory and nonverbal. A brain MRI at 12 yr of age was reported as normal.

Patient 5 is a 7-yr-old female with global developmental delay, intellectual disability, seizures, hypotonia, spasticity, dystonia, nonepileptic myoclonus, mild microcephaly (−2.8 SD), and cortical visual impairment. The patient was described as extremely irritable since birth and was noted to have hand stereotypies in infancy, although this later resolved. She came to medical attention at 4 mo of age with developmental delay and dystonia. The patient has drug-responsive generalized epilepsy with the most prominent seizure types being myoclonic seizures, and an EEG indicates photosensitivity. Her seizures are treated with clobazam. Levodopa treatment resulted in a moderate improvement in the patient's movement disorder, whereas trihexyphenidyl resulted in worsening. Two brain MRIs, the most recent at 6 yr of age, were reportedly normal. Although smaller than average, Patient 5 does not have failure to thrive. This patient has absent speech, but her receptive and nonverbal communication is felt to be a comparative strength.

### Brain MRI Review

**Figure 2.**
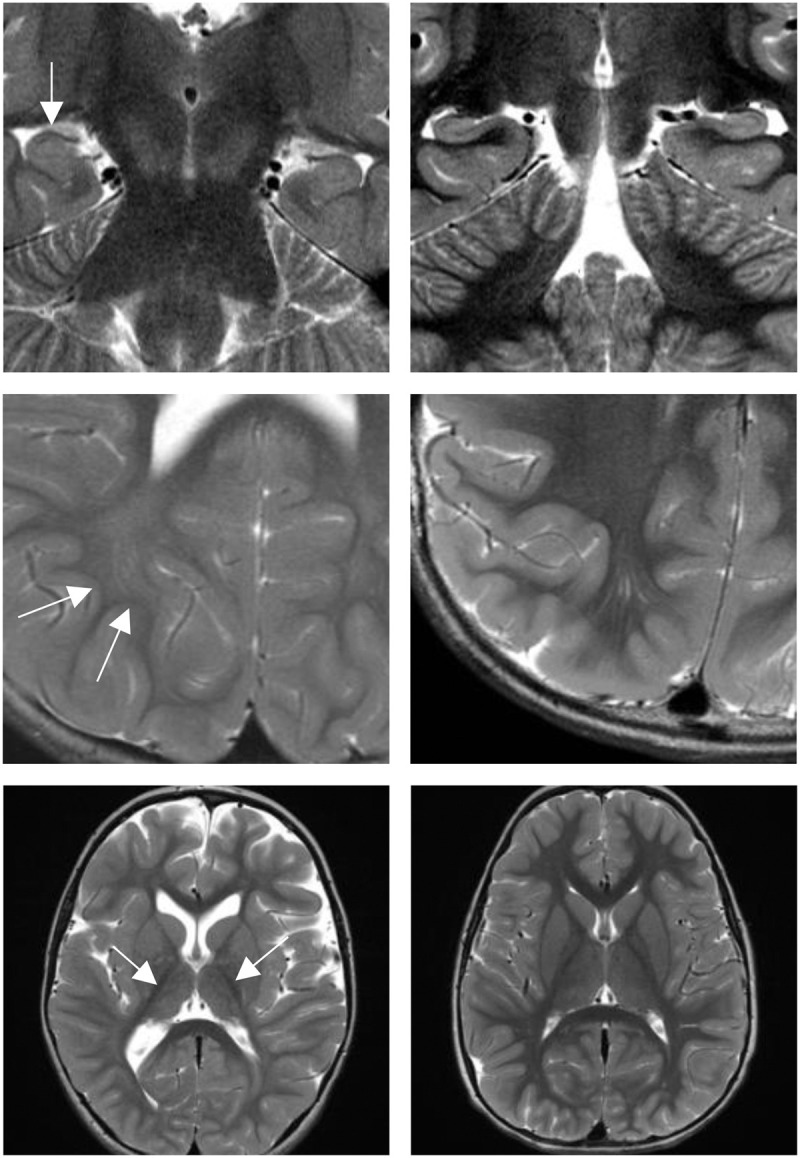
Brain MRI images: Patient 1 and control. Vertically oriented right hippocampus and blurring of the stratum radiatum in Patient 1 (*top left*, arrow) and age-matched control with normal hippocampi (*top right*). Attenuated subcortical U-fibers compared to white matter in Patient 1 (*middle left*, two white arrows) and age-matched normal control (*middle right*). Diminutive thalami in Patient 1 (*bottom left*, two white arrows) and normal thalami in age-matched control (*bottom right*).

Brain MRI images for all subjects were reviewed by the same neuroradiologist (P.A.C.) with 19 years of post-fellowship experience. The corpus callosum was normal in appearance in Patients 3, 4, and 5, whereas the posterior aspect of the corpus callosum was slightly thin and exaggeratedly downsloping in Patients 1 and 2. Patients 1, 4, and 5 displayed diminutive thalami, whereas the thalami were normal in Patients 2 and 3. In Patient 1 the right hippocampus was abnormally vertically oriented with blurring of the stratum radiatum. These hippocampal findings were not seen in the other four patients. However, in all but Patient 2, the image quality did not allow for assessment of the stratum radiatum. Patient 1 was noted to have patchy high T2 signal intensity in the subcortical and peri-atrial white matter of the posterior parieto-occipital lobes, a finding not seen in Patients 2, 3, or 4. The conspicuity of the subcortical U-fibers on the T2 weighted images was accentuated compared to the adjacent deep white matter in all but Patient 5. Patient 5 was noted to have many dilated perivascular spaces. (See Fig. 2 for Patient 1 brain MRI images.)

### Genomic Analyses and Variant Table

Each affected patient or family was tested by a different method, resulting in the identification of biallelic *UBA5* variants in each patient (Table 1; Supplemental Table S2). Average depth of coverage information for can be found in Supplemental Table S3.

**Table 1.** Variants table

Gene	Chromosome (GRCh37)	HGVS DNA reference	HGVS protein reference	Predicted effect	Previously reported	CADD score	dbSNP/dbVar ID	ClinVar ID
*UBA5*	Chr 3:g.132389876 C > T	c.562C > T	p.Arg188*	Nonsense	Yes	38	rs374052333	265749
*UBA5*	Chr 3:g.132390970 T > C	c.761T > C	p.Leu254Pro	Missense	No	31	N/A	1048516
*UBA5*	Chr 3:g.132394186 T > C	c.907T > C	p.Cys303Arg	Missense	No	29.3	rs1553770577	522846
*UBA5*	Chr 3:g.132394747 G > A	c.1111G > A	p.Ala371Thr	Missense	Yes	27.6	rs114925667	265745

#### Patient 1

Trio exome sequencing revealed compound heterozygous variants in *UBA5* (NM_024818.4). One variant, c.1111G > A (p.Ala371Thr), was reported as pathogenic and was inherited from the patient's father. The other variant, c.907T > C (p.Cys303Arg), was reported as a variant of uncertain significance and was inherited from the patient's mother. Although the p.Ala371Thr variant is the most frequently described variant to date in patients with EIEE44, the p.Cys303Arg variant has not been previously described in the literature and is not present in gnomAD, a large population database (https://gnomad.broadinstitute.org/). This variant is predicted to be damaging and probably damaging by SIFT and PolyPhen-2, respectively (http://genetics.bwh.harvard.edu/pph2, https://sift.bii.a-star.edu.sg/).

#### Patient 2

Quad genome sequencing of patient, parents, and an unaffected sibling revealed compound heterozygosity for *UBA5* variants, c.1111G > A (p.Ala371Thr) and c.562C > T (p.Arg188*), in the patient. The p.Ala371Thr variant was inherited from the father, and the p.Arg188* was inherited from the mother. The patient's unaffected sibling inherited only the p.Ala371Thr variant. The nonsense variant, p.Arg188*, is absent from the gnomAD database (https://gnomad.broadinstitute.org/) but has been reported in two patients with EIEE44, also in *trans* with p.Ala371Thr (Muona et al. 2016).

#### Patients 3 and 4

Sequencing of *UBA5* revealed compound heterozygosity for *UBA5* variants, c.1111G > A (p.Ala371Thr) and c.761T > C (p.Leu254Pro) in both affected siblings. The p.Ala371Thr variant was inherited from the father, and the p.Leu254Pro variant was inherited from the mother. This variant is absent from gnomAD (https://gnomad.broadinstitute.org/) and is predicted to be damaging and probably damaging by SIFT and PolyPhen-2, respectively (http://genetics.bwh.harvard.edu/pph2, https://sift.bii.a-star.edu.sg/).

#### Patient 5

Trio exome sequencing revealed the same variants seen in Patient 1, c.1111G > A (p.Ala371Thr) and (c.907T > C p.Cys303Arg). The p.Ala371Thr variant was inherited from the father, and the p.Cys303Arg variant was inherited from the mother.

### Functional Analyses

We examined the effects of UBA5^C303R^ and UBA5^L254P^ on the UFM1 system. In addition to these two novel variants, we tested the previously reported UBA5^A371T^ variant, which has comparable ability to wild type with regard to UFM1–UBA5 intermediate formation but is unable to transfer the activated UFM1 to UFC1. To do this we used a UBA5 mutant in which the active site, cysteine (Cys250), was substituted with serine (UBA5^C250S^). When the cysteine residue at the active site of E1 and E2 enzymes is replaced with serine, an *O*-ester bond instead of a thioester bond is formed with its respective modifier proteins, becoming stable even under reducing conditions (Komatsu et al. 2004). We compared the E1 activity to an active site variant, UBA5^C250A^, which prevents UFM1–UBA5 intermediate formation. To exclude the effect of endogenous UBA5, *UBA5*-deficient HEK293T cells were used (Muona et al. 2016).

**Figure 3.**
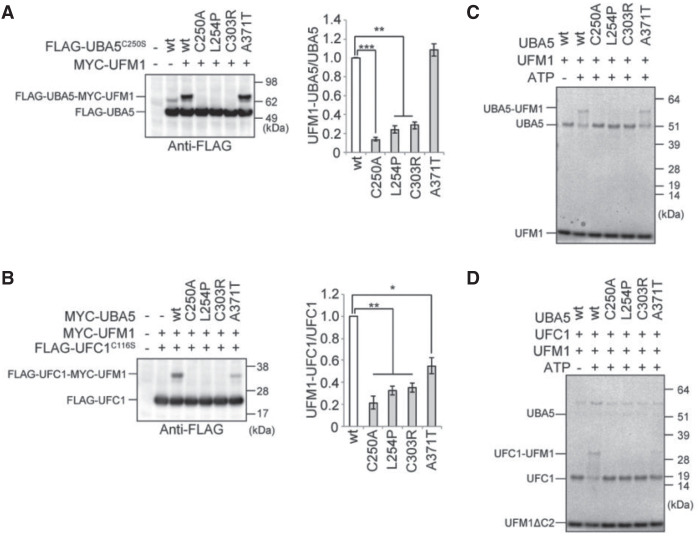
Immunoblot and in vitro thioester formation assays. (*A*,*B*) Immunoblot assay of UBA5 mutants in *UBA5*^−/−^ HEK293T cells. Indicated constructs (0.1 µg for UBA5 or UBA5 mutant, 0.5 µg for UFC1 or UFC mutant, and 2 µg for UFM1ΔC2) were expressed in *UBA5*-deficient HEK293T cells. Twenty-four hours after transfection, the cell lysates were subjected to immunoblot analysis with indicated antibodies. Bar graphs indicate the quantitative densitometric analyses of FLAG-UBA5-MYC-UFM1ΔC2 and FLAG-UFC1-MYC-UFM1ΔC2 intermediates relative to free FLAG-UBA5 and FLAG-UFC1, respectively. Statistical analyses were performed using the unpaired *t*-test (Welch test). The data represents the means ± SE of three separate experiments. (**) *P* < 0.01. (*C*,*D*) In vitro thioester formation assay of UFM1 by UBA5 (*C*) and of UFM1 by UFC1 (*D*). The assay was conducted as described in Methods. Data shown are representative of three separate experiments.

Initially, we tested the ability of these variants to form the UFM1–UBA5 intermediate in cells in an immunoblot assay. When UBA5^C250S^ was introduced into *UBA5*-knockout HEK293T cells, a stable intermediate was formed with both endogenous UFM1 and exogenous MYC–UFM1. We next analyzed UBA5 constructs with C250S and each of the missense variants. When UBA5^C250S/C303R^ or UBA5^C250S/L254P^ was introduced, very little intermediate formed, similar to the UBA5^C250A^ active site variant. In contrast, UBA5^C250S/A371T^ was not impaired in forming a UBA5–UFM1 intermediate (Fig. 3A). We next tested the ability of these UBA5 variants to transfer the activated UFM1 to UFC1 in an immunoblot assay using a UFC1 mutant in which the active site, cysteine (Cys116) was substituted with serine. When wild-type UBA5 was expressed together with UFC1^C116S^ in *UBA5*-deficient HEK293T cells, we clearly detected the intermediate of UFM1–UFC1^C116S^ with both endogenous UFM1 and exogenous MYC–UFM1. Formation of the UFM1–UFC1^C116S^ intermediate was abrogated when UBA5^C303R^ or UBA5^L254P^ was expressed together with UFC1^C116S^, again similar to the UBA5^C250A^ active site variant. UFC1–UFM1 intermediate formation was also decreased with UBA5^A371T^, although to a lesser degree (Fig. 3B).

An in vitro thioester assay demonstrated formation of the UBA5–UFM1 intermediate using wild-type UBA5, as well as UBA5^A371T^. Similar to UBA5^C250A^, the UBA5^C303R^, and UBA5^L245P^ variants did not result in UFM1 activation and formation of the UBA5–UFM1 intermediate (Fig. 3C). The thioester assay also revealed that, like active-site mutant UBA5^C250A^, UBA5^C303R^, UBA5^L245P^, and UBA5^A371T^ did not result in UFM1 transferring to UFC1 and formation of the intermediate (Fig. 3D). The variant p.Ala371Thr is located in the carboxy-terminal transthiolation domain, which is essential for transfer of UFM1 to UFC1 (Muona et al. 2016).

These results support previous findings showing that UBA5^A371T^ variant results in reduction in E2 but not E1 activity compared to wild-type UBA5. In addition, we conclude that two novel variants, UBA5^C303R^ and UBA5^L245P^, result in almost complete loss of E1 activity and a significant reduction of E2 activity.

## DISCUSSION

Our review of the now 24 reported individuals with EIEE44 from 17 families indicates four common findings: global developmental delay, axial hypotonia, abnormalities in motor control, and abnormal EEGs (for those in whom EEG was performed) (Table 2; Supplemental Table S1). Some features differ between and within families with EIEE44.

**Table 2.** Phenotype table

	Previously reported patients with EIEE44 (19)	Patient 1	Patient 2	Patient 3	Patient 4	Patient 5
Sex, age		F, 8 yr	F, 7 yr	M, 19 yr	F, 16 yr	F, 7 yr
Variants		p.Ala371Thr, p.Cys303R	p.Ala371T, p.Arg188*	p.Ala371Thr, p.Leu254Pro	p.Ala371Thr, p.Leu254Pro	p.Ala371Thr, p.Cys303R
Hypotonia	16/19 (84%)	+	+	+	+	+
Hypertonia/spasticity	13/19 (68%)	+	+	+	+	+
Movement disorder	15/19 (79%)	+	+	+	+	+
Seizures	16/19 (84%)	+	+	+	+	+
GDD/ID	18/19 (95%)	+	+	+	+	+
MRI abnormalities	16/16 (100%)	+	+	+	+	+
Microcephaly	17/19 (89%)	–	+	+	+	+
Failure to thrive	14/19 (74%)	+	+	+	+	–

(EIEE44) Early infantile epileptic encephalopathy-44, (GDD/ID) global developmental delay/intellectual disability, (MRI) magnetic resonance imaging, (+) is present, (–) is not present.

Seizures were reported in 21/24 (87.5%) subjects with EIEE44. Seizure types varied and included generalized and focal onset of motor and nonmotor seizures. Infantile spasms occurred in 13/24 (54%) individuals. Despite the disorder's name, the onset of seizures did not occur until childhood in two of the new patients described this report.

Abnormalities in motor control, most commonly dystonia, were noted in 20/24 (83%) of subjects with EIEE44. The overlap of motor and developmental phenotypes noted in our cohort and literature review add to the increasing understanding of epilepsy-movement phenotypes that span various genetic epileptic encephalopathies (Papandreou et al. 2020).

Several previous reports of EIEE44-associated *UBA5* variants included functional studies that were performed using the same methods presented in the current study (Komatsu et al. 2004).

Most variants were assessed for both E1 activity (the ability to form the UFM1–UBA5 intermediate) and E2 activity (the ability to form the UFM1–UFC1 intermediate, a process that also involves UBA5). The results, which can be viewed in the detailed variant table (Supplemental Table 2), show that the variants vary widely in severity. As in the current report, both Muona et al. and Colin et al. show that the common p.Arg371Thr variant's E1 activity is not significantly reduced compared to wild type, whereas its E2 activity is significantly reduced (*P* < 0.01 for prior studies, *P* < 0.05 for current study), making it one of the mildest variants reported to date (Colin et al. 2016; Muona et al. 2016). In the current study we also describe two novel variants, p.Leu254Pro and p.Cys303Arg, and show that both result in significant but incomplete reduction in both E1 and E2 activities compared to wild type (*P* < 0.01 for all).

Twelve of the 17 identified families with EIEE44 (70%), including all four families reported here, have the p.Ala371Thr variant in *trans* with another variant that has a more severe impact on UFMylation (Supplemental Table S2). The p.Ala371Thr variant is the most common disease-causing *UBA5* variant identified to date. The highest carrier frequency appears to be in the Finnish population, at 1 in 84 (https://gnomad.broadinstitute.org/variant/3-132394747-G-A). This likely represents a founder mutation in this population, which is known to have a high number of founder mutations (Norio et al. 1973). The carrier frequency in the non-Finnish European population is ∼1 in 200 (https://gnomad.broadinstitute.org/variant/3-132394747-G-A). Carriers of Latino, African, and “other” ancestry have also been identified, but at a much lower frequency (https://gnomad.broadinstitute.org/variant/3-132394747-G-A).

Four adult p.Ala371Thr homozygotes with no evidence of neurological disease have been identified from large Finnish and Icelandic population databases (Colin et al. 2016; Muona et al. 2016; Arnadottir et al. 2017). This finding implies that those with biallelic variants imparting an extremely mild effect on UFMylation are asymptomatic. Consistent with this hypothesis, the current study and two prior studies have shown that the p.Ala371Thr allele is hypomorphic and results in only slightly decreased enzyme activity (Colin et al. 2016; Muona et al. 2016; Ishimura et al. 2017).

*UBA5* knockout is embryonic lethal in mice because of severe anemia resulting from defective hematopoiesis (Tatsumi et al. 2011), and loss of function of this gene is also embryonic lethal in fruit flies (Duan et al. 2016). Consistent with this finding, no humans with biallelic loss of function *UBA5* variants have been reported to date. As no human patients with *UBA5*-related disease reported to date have been shown to have defective hematopoiesis, it can be hypothesized that residual UBA5 activity in human cases surviving to birth is sufficient for normal hematopoiesis (Muona et al. 2016).

The findings above point toward the existence of a lower threshold of UBA5 function below which survival to birth would not be possible as well as an upper threshold above which individuals are asymptomatic. Any level of UBA5 function between these two thresholds is anticipated to result in disease, potentially varying in severity by level of residual UBA5 function. Functional assessments of recurring and novel *UBA5* variants, such as the p.Cys303Arg and p.Leu254Pro variants reported here, are valuable to further elucidate this possible genotype–phenotype correlation.

In support of this theory, the most severely affected reported EIEE44 patient to date also appears to have had the most severe *UBA5* genotype reported to date. This patient, described by Mignon-Ravix and colleagues, presented with epileptic encephalopathy with burst suppression within hours of birth and died as a result of complications of status epilepticus at 16 days of age. The patient was found to be homozygous for the *UBA5* variant c.158A > T (p.Tyr53Phe), which was shown to have significantly reduced E1 activity (3.4%, *P* < 0.001) and E2 activity (6.8%, *P* < 0.001) compared to wild type (Mignon-Ravix et al. 2018; E. Liebau, email comm., November 24, 2020). No other clear genotype–phenotype correlation is evident within the 17 known EIEE44 families.

Although 17 of 19 families reported to date with biallelic *UBA5* mutations have the EIEE44 phenotype, the phenotypes of the two remaining families described by Duan et al. (2016) and Cabrera-Serrano et al. (2020) appear to be distinct (Supplemental Table S1). Other than the fact that neither of these two families carry the p.Ala371Thr variant, there is currently no identifiable genotype–phenotype correlation.

The family described by Duan and colleagues included two adult siblings with a progressive, childhood-onset cerebellar ataxia with normal intellect. Both had gait and limb ataxia, dysarthria, horizontal gaze nystagmus, cerebella atrophy, and cataracts. Neither had seizures. One of the siblings, who was more severely affected than the other, was shown to have a demyelinating sensory motor peripheral neuropathy (Duan et al. 2016).

One possible explanation for the difference in phenotype seen in the siblings reported by Duan and colleagues is that one or both variants are not pathogenic and that UBA5 dysfunction is thus not responsible for the phenotype. The siblings were found to have compound heterozygous variants in *UBA5*, a rare nonsense variant (c.736C > T, p.Arg246*) and a novel missense variant (c.760A > G, p.Lys310Glu). Duan and colleagues provided convincing evidence for the pathogenicity nonsense variant, showing that it localized mainly the nucleus, whereas the wild-type protein localized to the cytoplasm, failed to activate UFM1, and was significantly less stable than wild type. The evidence for the missense variant was less robust. In this case the mutant protein localized to the cytoplasm and did not result in significant reduction in UFM1 activation. The missense mutant protein was shown to be less stable than the wild-type protein, although the decrease in stability was much less dramatic than was seen for the nonsense variant (Duan et al. 2016). E1 and E2 activity were not assessed.

Most recently, Cabrera-Serrano and colleagues described a large consanguineous family in which five individuals had a severe congenital neuropathy that resulted in death in early infancy due to respiratory failure. The affected infants presented with areflexia, hypotonia, and progressive distal and proximal muscle weakness. They developed progressive breathing difficulties in the early weeks of life, and three developed tongue fasciculations. Brain and spine MRIs and EEGs, when performed, were unremarkable. Those tested were shown to have a sensory motor peripheral neuropathy (Cabrera-Serrano et al. 2020). Of note, none of the 24 identified EIEE44 patients are known to have had nerve conduction studies performed to assess for peripheral neuropathy.

The explanation for the apparently divergent phenotype in the family reported by Cabrera-Serrano and colleagues is not currently clear. The authors showed that, compared to wild-type, the homozygous missense variant in this family (c.31C > T, p.Arg11Trp), resulted in reduced UBA5 protein abundance and a significantly reduced ability to activate UFM1 (*P* < 0.05) (Cabrera-Serrano et al. 2020). Unlike all but one of the other *UBA5* variants described to date, this variant is intronic and unlikely to be damaging in the transcript that is most highly expressed in the brain, per GTEx (https://gtexportal.org/home/gene/UBA5). However, the same is true of the c.158A > T (p.Tyr53Phe) variant, which has been shown to have a severe effect and was seen in the homozygous state in the most severely affected patient with EIEE44 to date (Mignon-Ravix et al. 2018).

Our series showed abnormal MRI findings in all patients. Among these findings, of note, four out of the five patients showed an abnormal pattern of white matter involvement. On the T2 weighted images, the deep bifrontal and biparietal white matter exhibited abnormal, confluent, subtle high signal that resulted in relatively increased conspicuity of the subcortical U-fibers. We have observed this finding as a recognizable endophenotypic pattern in patients with mutations in genes associated with early infantile epileptic encephalopathy (P.A.C., pers. observation).

The identified symptoms of *UBA5*-related EIEE44 in humans have been almost exclusively neurological in nature, with one consistent exception. Most subjects, 18/24 (75%), presented with significant failure to thrive, often despite adequate caloric intake via G-tube. The two siblings reported by Duan et al. (2016) reportedly had markedly delayed growth in childhood. An explanation for this finding is currently unexplored. It is not yet known if the growth failure in this condition results from an intrinsic growth defect, an inability to process calories, or an increased caloric need. Only a small number of targets of UFMylation have been identified to date (Wei and Xu 2016). Thus, it is possible that the explanation for growth failure in *UBA5*-related encephalopathy lies in a yet unidentified target of UFMylation. Interestingly, the pituitary gland, which is important for growth, is listed as the highest expressing tissue in the Genotype-Tissue Expression (GTEx) Project (https://gtexportal.org/home/gene/UBA5). Of note, Patient 1 from the current study has been shown to have normal growth hormone levels.

In summary, we have described five new patients with EIEE44 from four unrelated families and provided functional evidence of pathogenicity for two novel *UBA5* variants, p.Cys303Arg and p.Leu245Pro. Including this report, there have now been 19 families described with biallelic *UBA5* variants, 17 with EIEE44 and two with different neurological phenotypes. Taken together, the data provides some information regarding an apparent genotype–phenotype. First, of the cases reported to date, the p.Ala371T variant has been present only in patients with the EIEE44 phenotype. Second, the patient with the most severe *UBA5* genotype was also the patient who presented with the most severe phenotype, dying of complications of status epilepticus in the first weeks of life (Mignon-Ravix et al. 2018).

The mechanisms by which biallelic *UBA5* variants cause the neurological and growth complications characteristic of EIEE44 and the observed phenotypic variability have not yet been elucidated. The identification of additional affected families, along with additional studies of the biochemical effects of various *UBA5* variants, may shed further light on the cause of phenotypic variability in individuals with biallelic *UBA5* variants. Consistent and detailed phenotyping and additional genotyping of affected individuals should contribute to this process.

## METHODS

### Patient Ascertainment

Patient 1 and Patient 2 were ascertained through their enrollment in the Undiagnosed Diseases Network (UDN) study. Patients 3 and 4, who are siblings, were ascertained through their application to the UDN study but were not enrolled in the study, as the *UBA5* variants had been previously identified through a prior research study. At the time of their initial identification, the *UBA5* variants had not yet been recognized to be the cause of the siblings’ condition. Patient 5 was identified through their enrollment in the Genetic Diagnosis in Childhood Onset Movement Disorders study.

### Sequencing

#### Patient 1

DNA extracted from blood was subjected to exome sequencing by Baylor Genetics Laboratories. Whole-exome capture was performed using biotin-labeled VCRome 2.1 in-solution exome probes. Sequencing was performed on the Illumina HiSeq platform for 100-bp paired end reads. Sequencing data was aligned to Human Genome Sequencing Center (HGSC) build 37, human reference genome 19 using the HGSC Mercury analysis pipeline, and variant calls were performed using Atlas-SNP and Atlas-Indel. All reported variants were confirmed by Sanger sequencing.

#### Patient 2

Genome sequencing of DNA isolated from blood was performed by HudsonAlpha Clinical Services Laboratory, LLC. Sequencing was performed on using Illumina HiSeq X flowcells. Reads were generated using Illumina's bcl2fastq, and data were aligned to HGSC build 37. Sequence analysis was performed using a custom platform called Codicem. All variants were confirmed by Sanger sequencing.

#### Patients 3 and 4

Next-generation sequencing (NGS) of *UBA5* was performed by Prevention Genetics. Genomic DNA was isolated from blood, barcoded, and enriched for the coding exons of targeted genes using hybrid capture technology. Prepared DNA libraries were sequenced using NGS technology. Following alignment, variants were detected in regions of at least 10× coverage. One hundred percent of coding regions and splicing junctions of *UBA5* had been sequenced with coverage of at least 10× and 20×, respectively, or by Sanger sequencing. Variants were interpreted manually using locus specific coverage for a heterozygous variant.

#### Patient 5

Library preparation and capture for exome sequencing was performed at the Kinghorn Centre for Clinical Genomics at the Garvan Institute of Medical Research using Agilent SureSelect Clinical Research Exome v2 (Agilent). Sequencing was performed on a HiSeqX (Illumina) to an average depth of coverage of 100× across captured regions. Alignments and variant calls were generated using SoftGenetics NextGene (version 2.4.1, SoftGenetics) to the February 2009 human genome assembly (GRCh37/hg19), and variant calls were restricted to coding regions and the canonical splice sites of an epileptic encephalopathy gene panel. Variants identified were then annotated using Alamut Batch (version 1.9, Interactive Biosoftware) and classified according to the American College of Medical Genetics (ACMG) criteria (Richards et al. 2015).

### Cell Culture

HEK293T cells were grown in Dulbecco's modified Eagle medium (DMEM) containing 10% fetal bovine serum, 5 U/mL penicillin, and 50 µg/mL streptomycin. *UBA5*-single and *UBA5 UFSP2*-double knockout HEK293T cells were used in this study (Muona et al. 2016; Ishimura et al. 2017).

### Immunoblot Analysis

Cells were lysed with ice-cold TNE buffer (10 mM Tris-Cl, pH 7.5, 1% Nonidet P-40, 150 mM NaCl, 1 mM ethylenediaminetetraacetic acid [EDTA], and protease inhibitors). The samples were separated using the NuPAGE system (Invitrogen) on 12% Bis-Tris gels in NuPAGE MOPS SDS Running Buffer and transferred to polyvinylidene difluoride (PVDF) membranes. Monoclonal antibody against FLAG was purchased from Medical & Biological Laboratories Co., Ltd., M185-3L. The immunoreactive bands were detected by LAS-4000 (GE Healthcare UK Ltd.). The quantitative densitometric analyses of FLAG–UBA5–MYC–UFM1ΔC2 relative to free FLAG–UBA5 and FLAG–UFC1–MYC–UFM1ΔC2 relative to free FLAG–UFC1 were carried out using Multi Gauge Version 3.2 Image software (Fuji Film). Statistical analysis was performed using an unpaired *t*-test (Welch test). The data represents the means ± SE of three separate experiments.

### In Vitro Thioester Formation Assay

An in vitro thioester formation assay was conducted as previously reported (Komatsu et al. 2004). Briefly, recombinant GST-UFM1ΔC2, GST-UBA5, GST-UBA5^C250A^, GST-UBA5^C303R^, GST-UBA5^A371T^, GST-UBA5^L254P^, and GST-UFC1 were produced in *Escherichia coli*, and recombinant proteins were purified by chromatography on Glutathione sepharose 4B (GE Healthcare UK Ltd). After digestion of GST by PreScission Protease (GE Healthcare UK Ltd), the recombinant proteins were dialyzed against 50 mM BisTris (pH 6.5), 100 mM NaCl, 10 mM MgCl_2_, and 0.1 mM DTT (reaction buffer). Thioester formation reactions contained reaction buffer with 0.8 µg UFM1ΔC2 and some of the following: 5 mM ATP, 0.08 µg (for UFC1–UFM1 thioester formation assay) or 0.8 µg (for UBA5–UFM1 thioester formation assay) UBA5 or UBA5 mutants, and 0.8 µg UFC1. Reactions were incubated for 5 min at 25°C and stopped by the addition of NuPAGE LDS Sample Buffer lacking reducing agent, followed by a 10-min incubation at 37°C, NuPAGE (4%–12% acrylamide gradient) and Coomassie brilliant blue staining. Data shown are representative of three separate experiments.

## The Members of the Undiagnosed Diseases Network

Maria T. Acosta,^15^ Margaret Adam,^15^,^16^ David R. Adams,^15^,^16^ Pankaj B. Agrawal,^17^ Mercedes E. Alejandro,^18^ Justin Alvey,^19^ Laura Amendola,^20^ Ashley Andrews,^19^ Euan A. Ashley,^21^ Mahshid S. Azamian,^18^ Carlos A. Bacino,^18^ Guney Bademci,^22^ Eva Baker,^15^ Ashok Balasubramanyam,^18^ Dustin Baldridge,^23^,^24^ Jim Bale,^19^ Michael Bamshad,^20^ Deborah Barbouth,^22^ Pinar Bayrak-Toydemir,^25^ Anita Beck,^20^ Alan H. Beggs,^17^ Edward Behrens,^26^ Gill Bejerano,^21^ Hugo J. Bellen,^38^ Jimmy Bennet,^20^ Beverly Berg-Rood,^20^ Jonathan A. Bernstein,^21^ Gerard T. Berry,^17^ Anna Bican,^27^ Stephanie Bivona,^22^ Elizabeth Blue,^20^ John Bohnsack,^19^ Carsten Bonnenmann,^15^ Devon Bonner,^21^ Lorenzo Botto,^19^ Brenna Boyd,^20^ Lauren C. Briere,^28^ Elly Brokamp,^27^ Gabrielle Brown,^29^ Elizabeth A. Burke,^15^,^16^ Lindsay C. Burrage,^18^ Manish J. Butte,^29^ Peter Byers,^20^ William E. Byrd,^30^ John Carey,^19^ Olveen Carrasquillo,^22^ Ta Chen Peter Chang,^22^ Sirisak Chanprasert,^20^ Hsiao-Tuan Chao,^18^ Gary D. Clark,^18^ Terra R. Coakley,^21^ Laurel A. Cobban,^31^ Joy D. Cogan,^27^ Matthew Coggins,^31^ F. Sessions Cole,^23^ Heather A. Colley,^32^ Cynthia M. Cooper,^28^ Heidi Cope,^33^ William J. Craigen,^18^ Andrew B. Crouse,^30^ Michael Cunningham,^20^ Precilla D'Souza,^15^ Hongzheng Dai,^18^ Surendra Dasari,^34^ Joie Davis,^15^,^16^ Jyoti G. Dayal,^32^ Matthew Deardorff,^26^ Esteban C. Dell'Angelica,^29^ Shweta U. Dhar,^18^ Katrina Dipple,^20^ Daniel Doherty,^20^ Naghmeh Dorrani,^29^ Argenia L. Doss,^32^ Emilie D. Douine,^29^ David D. Draper,^15^ Laura Duncan,^27^ Dawn Earl,^20^ David J. Eckstein,^32^ Lisa T. Emrick,^18^ Christine M. Eng,^35^ Cecilia Esteves,^36^ Marni Falk,^26^ Liliana Fernandez,^21^ Carlos Ferreira,^15^ Elizabeth L. Fieg,^31^ Laurie C. Findley,^32^ Paul G. Fisher,^21^ Brent L. Fogel,^29^ Irman Forghani,^22^ William A. Gahl,^15^,^16^ Ian Glass,^20^ Bernadette Gochuico,^15^,^16^ Rena A. Godfrey,^15^ Katie Golden-Grant,^20^ Alica M. Goldman,^18^ Madison P. Goldrich,^32^ David B. Goldstein,^37^ Alana Grajewski,^22^ Catherine A. Groden,^15^ Irma Gutierrez,^29^ Sihoun Hahn,^20^ Rizwan Hamid,^27^ Neil A. Hanchard,^18^ Athena Hantzaridis,^36^ Kelly Hassey,^26^ Nichole Hayes,^24^ Frances High,^28^ Anne Hing,^20^ Fuki M. Hisama,^20^ Ingrid A. Holm,^17^ Jason Hom,^21^ Martha Horike-Pyne,^20^ Alden Huang,^29^ Yong Huang,^21^ Laryssa Huryn,^15^,^16^ Rosario Isasi,^22^ Fariha Jama,l8 Gail P. Jarvik,^20^ Jeffrey Jarvik,^20^ Suman Jayadev,^20^ Lefkothea Karaviti,^18^ Jennifer Kennedy,^27^ Dana Kiley,^23^ Shilpa N. Kobren,^36^ Isaac S. Kohane,^36^ Jennefer N. Kohler,^21^ Deborah Krakow,^29^ Donna M. Krasnewich,^32^ Elijah Kravets,^21^ Susan Korrick,^31^ Mary Koziura,^27^ Joel B. Krier,^31^ Seema R. Lalani,^18^ Byron Lam,^22^ Christina Lam,^20^ Grace L. LaMoure,^32^ Brendan C. Lanpher,^34^ Ian R. Lanza,^34^ Lea Latham,^15^,^16^ Kimberly LeBlanc,^36^ Brendan H. Lee,^18^ Hane Lee,^29^ Roy Levitt,^22^ Richard A. Lewis,^18^ Sharyn A. Lincoln,^17^ Pengfei Liu,^35^ Xue Zhong Liu,^22^ Nicola Longo,^24^ Sandra K. Loo,^29^ Joseph Loscalzo,^31^ Richard L. Maas,^31^ John MacDowall,^15^,^16^ Ellen F. Macnamara,^15^ Calum A. MacRae,^31^ Valerie V. Maduro,^15^ Bryan C. Mak,^29^ May Christine V. Malicdan,^15^,^16^ Laura A. Mamounas,^32^ Teri A. Manolio,^32^ Rong Mao,^25^ Kenneth Maravilla,^20^ Thomas C. Markello,^15^ Ronit Marom,^18^ Gabor Marth,^19^ Beth A. Martin,^21^ Martin G. Martin,^29^ Julian A. Martínez-Agosto,^29^ Shruti Marwaha,^21^ Jacob McCauley,^22^ Allyn McConkie-Rosell,^33^ Alexa T. McCray,^36^ Elisabeth McGee,^29^ Heather Mefford,^20^ J. Lawrence Merritt,^20^ Matthew Might,^30^ Ghayda Mirzaa,^20^ Eva Morava,^34^ Paolo M. Moretti,^18^ Deborah Mosbrook-Davis,^15^,^16^ John J. Mulvihill,^32^ David R. Murdock,^18^ Anna Nagy,^36^ Mariko Nakano-Okuno,^30^ Avi Nath,^15^ Stan F. Nelson,^29^ John H. Newman,^27^ Sarah K. Nicholas,^18^ Deborah Nickerson,^20^ Shirley Nieves-Rodriguez,^29^ Donna Novacic,^15^ Devin Oglesbee,^34^ James P. Orengo,^18^ Laura Pace,^19^ Stephen Pak,^24^ J. Carl Pallais,^31^ Christina GS. Palmer,^29^ Jeanette C. Papp,^29^ Neil H. Parker,^29^ John A. Phillips, III,^27^ Jennifer E. Posey,^18^ Lorraine Potocki,^18^ Bradley Power,^15^,^16^ Barbara N. Pusey,^15^ Aaron Quinlan,^19^ Wendy Raskind,^20^ Archana N. Raja,^21^ Deepak A. Rao,^31^ Genecee Renteria,^29^ Chloe M. Reuter,^21^ Lynette Rives,^27^ Amy K. Robertson,^27^ Lance H. Rodan,^17^ Jill A. Rosenfeld,^18^ Natalie Rosenwasser,^20^ Francis Rossignol,^15^,^16^ Maura Ruzhnikov,^21^ Ralph Sacco,^22^ Jacinda B. Sampson,^21^ Susan L. Samson,^18^ Mario Saporta,^22^ Judy Schaechter,^22^ Timothy Schedl,^24^ Kelly Schoch,^33^ Daryl A. Scott,^18^ C. Ron Scott,^20^ Vandana Shashi,^33^ Jimann Shin,^24^ Rebecca Signer,^29^ Edwin K. Silverman,^31^ Janet S. Sinsheimer,^29^ Kathy Sisco,^23^ Edward C. Smith,^33^ Kevin S. Smith,^21^ Emily Solem,^27^ Lilianna Solnica-Krezel,^24^ Ben Solomon,^15^,^16^ Rebecca C. Spillmann,^33^ Joan M. Stoler,^17^ Jennifer A. Sullivan,^33^ Kathleen Sullivan,^26^ Angela Sun,^20^ Shirley Sutton,^21^ David A. Sweetser,^28^ Virginia Sybert,^20^ Holly K. Tabor,^21^ Amelia L. M. Tan,^36^ Queenie K.-G. Tan,^33^ Mustafa Tekin,^22^ Fred Telischi,^22^ Willa Thorson,^22^ Audrey Thurm,^15^,^16^ Cynthia J. Tifft,^15^,^16^ Camilo Toro,^15^ Alyssa A. Tran,^18^ Brianna M. Tucker,^21^ Tiina K. Urv,^32^ Adeline Vanderver,^26^ Matt Velinder,^19^ Dave Viskochil,^19^ Tiphanie P. Vogel,^18^ Colleen E. Wahl,^15^ Melissa Walker,^28^ Stephanie Wallace,^20^ Nicole M. Walley,^33^ Chris A. Walsh,^17^ Jennifer Wambach,^23^ Jijun Wan,^29^ Lee-kai Wang,^29^ Michael F. Wangler,^38^ Patricia A. Ward,^35^ Daniel Wegner,^23^ Mark Wener,^20^ Tara Wenger,^20^ Katherine Wesseling Perry,^29^ Monte Westerfield,^39^ Matthew T. Wheeler,^21^ Jordan Whitlock,^30^ Lynne A. Wolfe,^15^,^16^ Jeremy D. Woods,^29^ Shinya Yamamoto,^38^ John Yang,^15^,^16^ Muhammad Yousef,^15^,^16^ Diane B. Zastrow,^21^ Wadih Zein,^15^,^16^ Chunli Zhao,^21^ Stephan Zuchner,^22^

## ADDITIONAL INFORMATION

### Data Deposition and Access

The deidentified sequencing data and phenotype data for Patient 1 and Patient 2 are available in the National Center for Biotechnology (NCBI) database of Genotypes and Phenotypes (dbGap; http://www.ncbi.nlm.nih/gov/gap) under the accession number phs001232.v1.p1. Consent for Patients 3, 4, and 5 did not allow for upload to dbGap. The variants identified in the five patients have been submitted to ClinVar (https://www.ncbi.nlm.nih/gov/clinvar/) and can be found under the accession numbers SCV000746680.3, SCV000746628.4, SCV00746679.1, SCV000746629.2, SCV001548168, SCV001548169, and SCV001548170.

### Ethics Statement

Patients 1 and 2 were enrolled in the Undiagnosed Diseases Network study, National Institutes of Health (NIH) protocol 15-HG-0130. Patient 5 was enrolled in the Genetic Diagnosis in Childhood Onset Movement Disorders study, Sydney Children’s Hospitals Network, HREC/18/SCHN/433. For both studies, written informed consent was obtained for probands and parents and includes permission for publication of findings in medical/research journals. Explicit written consent for publication of this manuscript was obtained for all five patients.

### Acknowledgments

We sincerely thank our five patients and their families for their participation.

### Author Contributions

L.C.B. and C.A.R. wrote the manuscript (others helped with revisions, etc.). P.A.C. reviewed the brain MRI images for all five patients. D.A.S, F.A.H., M.A.W., N.S., and L.C.B. contributed to the clinical diagnosis and description of Patient 1. J.A.P., M.E.K., and E.B. contributed to the clinical diagnosis and description of Patients 2, 3, and 4. S.S.M., R.C.D., and L.G.R. contributed to the clinical diagnosis and description of Patient 5. M.K., R.I., and Y.I. performed the functional analysis. All authors approved the manuscript before submission.

### Funding

Research reported in this manuscript was supported by the NIH Common Fund, through the Office of Strategic Coordination/Office of the NIH Director under award numbers U01HG007690 (L.C.B, C.J.C, C.C., F.A.H., C.A.R., D.A.S., M.A.W.) and U01HG007674 (E.B., M.E.K., J.A.P.). The content is solely the responsibility of the authors and does not necessarily represent the official views of the National Institutes of Health. The Hill Family Fund for the Diagnosis and Management of Rare and Undiagnosed Diseases at Mass General and a donation by Seta and Douglas Atamian also supported this work. R.I. is supported by Grant-in-Aid for Early-Career Scientists (20K16161). Y.I. is supported by Grant-in-Aid for Scientific Research (C) (20K06644). M.K. is supported by Grant-in-Aid for Scientific Research on Innovative Areas (19H05706), Grant-in-Aid for Scientific Research (B) (18H02611), a Japan Society for the Promotion of Science (an A3 foresight program, to M.K.), and the Takeda Science Foundation (to M.K.). S.M. is supported a project grant from the Cerebral Palsy Alliance (PG01217).

### Competing Interest Statement

The authors have declared no competing interest.

## Supplementary Material

Supplemental Material
