# Supplementary material for: A description of novel variants and review of phenotypic spectrum in UBA5-related early epileptic encephalopathy
Source: Cold Spring Harb Mol Case Stud. 2021 Jun;7(3):a005827. doi: 10.1101/mcs.a005827 (PMC8208045; doi:10.1101/mcs.a005827)
Supplement: Supplemental Material [file supp_7_3_a005827__DC1.html]

A description of novel variants and review of phenotypic spectrum in UBA5-related early epileptic encephalopathy — Supplemental Material 

# A description of novel variants and review of phenotypic spectrum in *UBA5*-related early epileptic encephalopathy

## Supplemental Material

- Supplemental\_Table\_S1.xlsx
- Supplemental\_Table\_S2.xlsx
- Supplemental\_Table\_S3.xlsx
